# Microscopic Image of Leishman-Donovan Bodies in Bone Marrow Aspirate Smear of Patient Suffering from Unexplained Intermittent Low-Grade Fever and Cough

**DOI:** 10.4274/tjh.2016.0285

**Published:** 2017-08-02

**Authors:** Kuenzang Dorji, Tashi Tobgay, Rixin Jamtsho, Puja Devi Samal, Pratap Rai

**Affiliations:** 1 Jigme Dorji Wangchuck National Referral Hospital, Clinic of Pathology and Laboratory Medicine, Hematology Unit, Thimphu, Bhutan; 2 Ministry of Health, Quality Assurance Standardizing Division, Thimphu, Bhutan

**Keywords:** Leishman-Donovan body, Bone marrow aspirate smear, Microscopic image

A 24-year-old male from Wangdue Phodrang district, Bhutan, presented with a history of unexplained intermittent low-grade fever and cough for 8 months. He also complained of weight loss, abdominal discomfort, and one episode of hemoptysis and convulsion. He was a cow herder by profession. A computed tomography scan showed hepatosplenomegaly (liver: 22 cm, spleen: 27 cm) with mesenteric lymphadenopathy. Biochemical evaluation showed elevated alkaline phosphatase (1096 IU/L), lactate dehydrogenase (330 IU/L), and C-reactive protein (40.8 mg/dL) and reverse albumin/globulin ratio. Complete blood count showed pancytopenia confirmed by peripheral smear. In addition, left shift of neutrophils and giant platelets were observed. Erythrocyte sedimentation rate (26 mm/h) was increased. Prothrombin time (20 s) and activated partial thromboplastin time (51 s) were prolonged. The bone marrow aspirate smear revealed intracellular and extracellular Leishman-Donovan bodies (Figure 1). In view of clinical and laboratory features, a diagnosis of visceral leishmaniasis was made [1,2,3]. However, the results of the standart devation BIOLINE Leishmania Ab rapid test kit (Standard Diagnostics Inc., Korea) directed against the rK39 antigen were negative. As per the protocol of the Government of India, parasitological diagnosis is the gold standard, which was observed in the bone marrow in this case. Serological diagnosis, which can be 95% sensitive and specific, is used for supportive evidence and fieldwork.

## Figures and Tables

**Figure 1:**
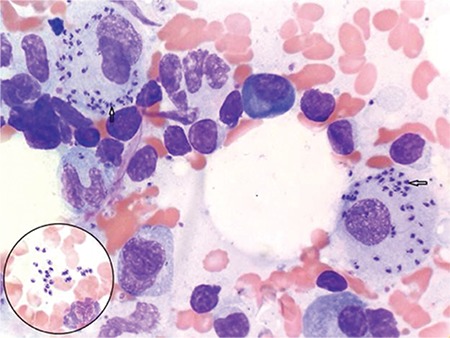
Bone marrow aspirate smear showing intracellular and extracellular (inset) Leishman-Donovan bodies. Leishman-Giemsa stain, 1000^x^.

## References

[1] Varma N, Naseem S (2010). Hematologic changes in visceral leishmaniasis/kala azar. Indian J Hematol Blood Transfus.

[2] Chakrabarti S, Sarkar S, Goswami BK, Sarkar N, Das S (2013). Clinico-hematological profile of visceral leishmaniasis among immunocompetent patients. Southeast Asian J Trop Med Public Health.

[3] Sarkari B, Naraki T, Ghatee MA, Abdolahi KS, Davami MH (2016). Visceral leishmaniasis in southwestern Iran: a retrospective clinico-hematological analysis of 380 consecutive hospitalized cases (1999-2014). PLoS One.

